# Data for a full-factorial cross-backend benchmark of runtime burden allocation in structured LLM routing

**DOI:** 10.1016/j.dib.2026.113321

**Published:** 2026-10-09

**Authors:** Hanlin Zhou, HuahYong Chan

**Affiliations:** a School of Computer Sciences, Universiti Sains Malaysia, 11800 USM, Pulau Pinang, Malaysia; b Xiamen Institute of Software Technology, Xiamen 361024, China

**Keywords:** Structured LLM routing, Full-factorial benchmark, Function calling, JSON schema, Runtime burden allocation, Cross-backend evaluation, Reproducible dataset, Mendeley data

## Abstract

This Data in Brief article describes a Mendeley Data package for a full-factorial benchmark of runtime burden allocation in structured large language model (LLM) routing. The benchmark crosses three backend families, four runtime-packaging modes, two constraint settings, and two transport settings, producing 48 deployment combinations and 15,552 planned request positions. The package separates four data layers: the full-factorial design, a denominator-only planned request index, the first persistent code-generated benchmark reports archived in F.zip, and processed aggregate statistical tables derived from the archived reports. It also contains runtime-mode definitions, manuscript-facing CSVs, a reusable request-level schema, figures, metadata, checksums, and lightweight validation scripts. The package supports structured-generation auditing, function-calling comparison, local-reconstruction baselines, benchmark teaching, and secondary analysis without requiring provider credentials; rerunning the hosted backend calls requires separate credentials and current provider access.

Specifications Table

**Table:** 

Subject	Computer Science
Specific subject area	Structured LLM routing; JSON-schema validation; function-calling benchmark; runtime burden allocation.
Type of data	Original code-generated benchmark report artifacts; denominator-only planned-event index; CSV tables; JSON schema; Python scripts; PNG figures; Markdown/TXT metadata.
Data collection	The harness crossed backend family, runtime package, constraint setting, and transport setting. Each of the 48 combinations used the same 324-request pool (36 request instances x 3 difficulty strata x 3 concurrency levels). The 15,552-row index records planned positions and factor labels only; aggregate outcomes were derived separately from the archived benchmark reports.
Data source location	Institution: Universiti Sains Malaysia, Pulau Pinang, Malaysia; Xiamen Institute of Software Technology, Xiamen, China. The dataset was generated and curated as a computational software-benchmark dataset.
Data accessibility	Repository name: Mendeley Data. Data identification number: gyb3446fkv. Version: 1. DOI: 10.17632/gyb3446fkv.1. Direct URL to data: https://data.mendeley.com/datasets/gyb3446fkv/1. Instructions for accessing these data: the dataset is publicly accessible without access controls. Reuse licence: data and metadata are released under CC BY 4.0; reference code is released under the MIT License.
Related research article	H. Zhou and H.Y. Chan, Runtime Burden Allocation for Structured LLM Routing in Agentic Expert Systems: A Full-Factorial Cross-Backend Methodology, arXiv:2604.01235, 2026.

## 1 Value of the Data

• LLM systems researchers can use the balanced design to compare how the same structured-routing task behaves across backend families, runtime packages, constraint profiles, and transport settings while the request denominator remains fixed.
• Benchmark developers can use the 48-row design matrix and stable combination_id field, which uniquely identifies each factorial deployment combination, to extend the experiment, verify the planned denominator, and retain machine-readable factor labels across new runs.
• As a concrete reuse example, a researcher evaluating a fifth runtime package can add 12 combinations (3 backends x 2 constraint settings x 2 transport settings). Reusing the same 324-request pool would add 3,888 planned request positions, and the resulting summaries can be joined to the released design through new combination identifiers for direct comparison with MJ, SJ, MJS, and MCLR.
• Software engineers can compare format compliance, routing accuracy, state retention, latency, token burden, and failure classes to screen backend-package compatibility before selecting a structured-control interface. For example, users can load data/processed/backend_mode_cell_means.csv to compare MJ/SJ with MCLR for a chosen backend and examine routing-accuracy, state-retention, latency, and token-burden trade-offs without rerunning hosted APIs.
• Reproducibility auditors can distinguish the design matrix, the denominator-only planned index, the original generated report artifacts, and the processed aggregate layer, and can verify file integrity through the schema notes, inventory, checksums, and validation script.
• Instructors can use the package to demonstrate full-factorial benchmark design, data provenance, sanitization boundaries, and the difference between offline validation and a credentialed API rerun.
• Model-version auditors can repeat selected cells with a later hosted-model snapshot while retaining the archived factor labels, prompt pool, and metric definitions, enabling a documented longitudinal comparison without overwriting the 22 March 2026 benchmark.

## 2 Background

Structured LLM routing systems increasingly rely on runtime packages that distribute burden between model-side structured generation, API transport behavior, deterministic local reconstruction, and post-hoc validation. For this reason, the dataset is organized around machine-readable factor labels, a denominator-only planned request index, reusable schema documentation, and aggregate outcome tables rather than only final accuracy values [1].

The data publication uses a persistent-data packaging style so that the factorial design, the denominator-only planned index, the original generated-report artifacts, and the processed statistical layer can be distinguished and cited independently of the article. This design is consistent with FAIR data principles and with data-citation guidance that emphasizes persistent identification, access, and reuse [2,3].

The package also reflects the practical heterogeneity of structured-output interfaces. Current OpenAI documentation describes schema-constrained structured model outputs, while Gemini documentation distinguishes structured outputs and function calling as separate interface patterns [[4], [5], [6]].

Because the benchmark is factorial, the design matrix is retained as a first-class data object rather than only as a narrative method description. This follows experimental-design practice in which factor levels, combinations, and response variables should remain auditable after aggregation [7].

The inclusion of the first persistent code-generated reports responds to data-documentation concerns raised for reusable datasets: users can distinguish generated source artifacts, design records, processed outputs, and manuscript previews rather than receiving only final tables [8,9].

## 3 Data Description

The Mendeley Data package [10] is organized as the folder DIB_Runtime_Burden_Mendeley_Dataset and contains four related but non-equivalent data layers. The 48-row design matrix defines the deployment combinations. The planned-event index expands that design to 15,552 request positions but stores no observed provider outcomes. F.zip preserves the first persistent code-generated benchmark reports. The processed CSV files contain aggregate metrics and failure classifications derived separately from those reports. The design matrix defines the planned-index path, while hosted benchmark execution produces generated reports for separate aggregate processing. Sanitized data, manuscript-facing tables and figures, metadata, schema documentation, checksums, and validation scripts are then packaged for release. Fig. 1 summarizes the relationships among these repository components.

**Fig. 1:**
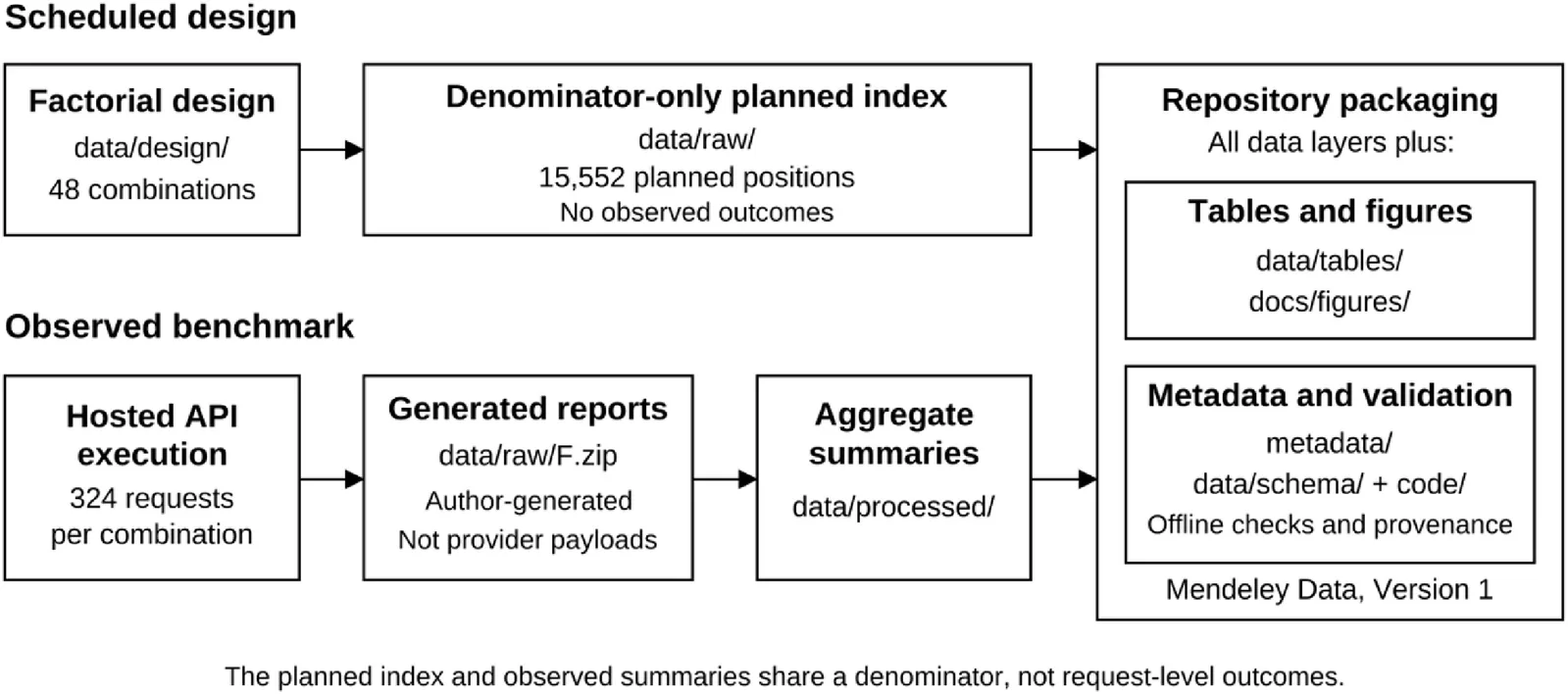
Relationships among the principal repository components. The scheduled-design path links the factorial design to the denominator-only planned index. The observed-benchmark path links hosted execution to author-generated report artifacts and separately derived aggregate summaries. Both paths are packaged with manuscript-facing tables and figures, metadata, schema documentation, and offline validation resources. The planned index contains no observed outcomes, and the generated reports are not provider-native response payloads.

Recommended first-use order: inspect (1) the 48-row design matrix, (2) the denominator-only planned index, (3) the archived generated reports, and (4) the processed aggregate tables; then use Table 9 to locate the source file for a specific table, figure, or implementation setting.

Table 1 lists the principal data objects and clarifies which files describe the planned design, which files preserve generated report artifacts, and which files contain processed aggregate outcomes.

**Table 1:** Principal data objects in the Mendeley Data package and their roles.

Mendeley Data package path	Data object	Use in the data article
README.md	Dataset-level overview	Explains scope, folder structure, Mendeley dataset information, and reuse commands.
data/raw/F.zip	Original generated-report archive	Archive of the first persistent author-generated benchmark reports and generation code; it is not a collection of provider-native API payloads.
data/raw/F_zip_original_raw_outputs/	Extracted generated report artifacts	Location for details_20260322_165132, detail_*.docx, summary/final reports, and detail.py, preserved as generated without manuscript-level rewriting.
data/raw/planned_request_event_index_15552.csv	Denominator-only planned request index	Enumerates 15,552 planned request positions and their factor labels; it contains no provider responses or request-level performance outcomes.
data/design/full_factorial_design_48.csv	Full-factorial design matrix	Lists 48 combinations across backend, runtime mode, constraints, and transport.
data/processed/*.csv	Processed statistical layer	Contains aggregate metrics and failure classifications derived separately from the archived benchmark reports, including cell means, ANOVA tables, contrasts, workflow completion, and failure taxonomy.
data/tables/*.csv	Manuscript-facing and processed tables	Mirrors manuscript-facing tables and processed statistical-layer CSVs for Mendeley reuse.
docs/figures/*.png	Architecture and result figures	Stores the architecture figure, result figure, and supporting figure files.
metadata/checksums_sha256.csv	Integrity checksums	Allows local verification of file contents after download.
code/reproduce_summary_tables.py	Offline validation script	Checks design size, planned-event count, summary cells, and failure totals; it does not rerun hosted APIs or reconstruct provider-native responses.
data/schema/request_level_event_schema.json	Reusable request-level outcome schema	Documents the intended structure of a future fully sanitized outcome record; it is not the schema of the denominator-only planned index.
LICENSE.txt	Reuse licences	CC BY 4.0 for the released data and metadata; MIT License for the reference code.

Table 2 gives the expected dimensions used for quick validation. The names in the table follow the machine-readable labels used in the released design files. Terminology note: the companion methodology article used “strict” and “relaxed” for the same constraint profiles recorded as “limited” and “unlimited,” respectively, in the released machine-readable files. This data article uses the archived labels throughout and standardizes the phrase “processed statistical layer” in place of the earlier wording “previous statistical layer.”

**Table 2:** Benchmark dimensions and expected counts for validation.

Dimension	Count	Definition
Backend families	3	Gemini, OpenAI, Llama
Runtime modes	4	MJ, SJ, MJS, MCLR
Constraint settings	2	Limited and unlimited
Transport settings	2	Stream and non-stream
Deployment combinations	48	3 × 4 × 2 × 2
Planned events per combination	324	36 request instances x 3 difficulty strata x 3 concurrency levels
Total planned request events	15,552	48 × 324
Backend-mode summary cells	12	3 backends x 4 modes
Original generated-report archive	F.zip	First persistent author-generated benchmark reports and generation code
Route labels	4	chat, task, dev, doc

Table 3 defines the named runtime packages. The package name and the execution-time transport factor are distinct: stream/non-stream transport is overlaid separately on all four named packages in the factorial design.

**Table 3:** Definitions and operational meanings of the four runtime packages.

Mode	Expanded name	Operational meaning
MJ	Minimal JSON	Low-budget direct JSON package; the model emits the final canonical JSON record.
SJ	Structured JSON	Higher-budget direct JSON package; the model emits the final canonical JSON record.
MJS	JSON package with historical streaming label	Historical package label retained for continuity; stream/non-stream transport is overlaid separately in the factorial design.
MCLR	Minimal compact local reconstruction	The model emits a compact intermediate code, which deterministic client logic expands into the canonical JSON record.

Table 4 reproduces the six entries in metadata/metric_dictionary.csv. The failure taxonomy is documented separately in Table 6 because it is an aggregate classification output rather than a field in the denominator-only planned-event index.

**Table 4:** Definitions of the six processed performance metrics.

Metric or field	Description
format_compliance_pct	Whether the emitted control record was parseable and schema-valid after any declared deterministic reconstruction.
routing_accuracy_pct	Whether the emitted route label matched the frozen benchmark route key.
state_retention_pct	Whether state-sensitive or interrupted cases were handled correctly where a state target was defined.
p50_latency_ms	Median full-response latency; downstream dispatch cannot proceed until the complete control record is available.
tokens_total	Total model-token consumption associated with the completed requests in the summarized cell.
workflow_lower_bound_completion_pct	Conservative lower-bound completion proxy derived from FC, RA, and SR, with SR omitted where no state target exists.

Fig. 2 visualizes the complete 12-cell backend-by-mode comparison for format compliance, routing accuracy, and state retention. The underlying source table is included in docs/figure_data/result_backend_mode_metrics_source.csv.

**Fig. 2:**
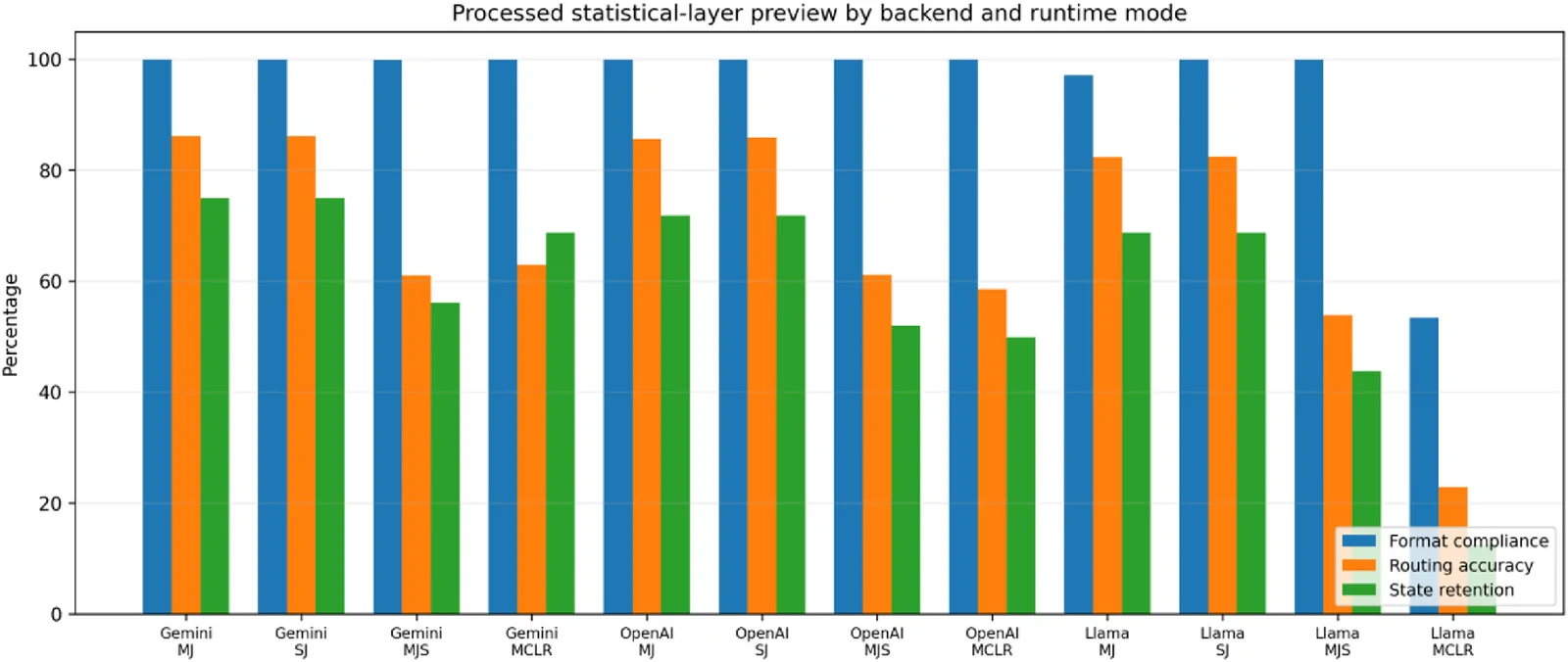
Complete backend-by-mode comparison of format compliance, routing accuracy, and state retention.

Table 5 reports all 12 backend-by-mode cells from backend_mode_cell_means.csv so that the article is self-contained. The released CSV also retains descriptive half-width fields for variation across the four constraint/transport subconditions within each cell.

**Table 5:** Complete backend-by-mode summary of format compliance, routing accuracy, state retention, median latency, and total token consumption.

Backend	Mode	Format compliance (%)	Routing accuracy (%)	State retention (%)	p50 latency (ms)	Tokens total
Gemini	MJ	100.00	86.11	75.00	1153.28	126,098
Gemini	SJ	100.00	86.11	75.00	1161.81	126,098
Gemini	MJS	99.92	61.03	56.08	1190.01	60,133
Gemini	MCLR	100.00	62.96	68.75	1014.09	45,771
OpenAI	MJ	100.00	85.65	71.88	1043.71	115,810
OpenAI	SJ	100.00	85.88	71.88	1041.49	115,816
OpenAI	MJS	100.00	61.11	51.91	1032.27	51,950
OpenAI	MCLR	100.00	58.49	49.83	661.51	40,036
Llama	MJ	97.14	82.33	68.75	231.89	130,459
Llama	SJ	100.00	82.41	68.75	235.52	133,457
Llama	MJS	100.00	53.85	43.75	215.33	69,232
Llama	MCLR	53.40	22.84	12.50	174.31	55,823

The Llama/MCLR cell is best read as a backend-package failure regime rather than as an isolated random outlier. It combines the lowest format compliance (53.40%), routing accuracy (22.84%), and state retention (12.50%) with the lowest median latency (174.31 ms). The route-level summary indicates selective fragility: developer-route accuracy falls to 0.00%, while conversational routing remains materially higher. The compact MCLR pathway can reconstruct canonical JSON only after the backend emits a usable intermediate code; deterministic reconstruction cannot recover an incorrect, incomplete, or malformed semantic code. Because MCLR bundles compact emission, a low output budget, and local reconstruction, the released data identify the incompatibility but do not isolate a single micro-level cause.

Table 6 reports aggregate outcome classifications derived from the archived benchmark reports. The counts sum to 15,552 because the processed taxonomy covers the same benchmark denominator, but these classifications are not stored row by row in planned_request_event_index_15552.csv.

**Table 6:** Overall failure taxonomy: event counts and percentages across the 15,552-request benchmark.

Event class	Count	Percentage of total events
complete_without_failure	14910	95.87
json_parse_error	523	3.36
schema_invalid_output	100	0.64
http_400_error	18	0.12
rate_limit_event	1	0.01

Table 7 separates the original generated-report layer from the denominator-only planned-event index. In this article, raw means the first persistent author-generated artifacts emitted by the benchmark/reporting code, not provider-native API payloads. The archived .docx copies were preserved as generated, without manual post-generation editing or manuscript-level rewriting.

**Table 7:** Provenance and roles of the original generated-report artifacts and denominator-only planned-event index.

Object or pattern	Layer	Role
F.zip	Original generated-report archive	Archive of the first persistent author-generated benchmark reports and generation code; not provider-native responses.
details_20260322_165132 / detail_*.docx	Code-generated detail report artifacts	First persistent detail reports emitted by the benchmark/report-generation code and archived without post-generation editing.
summary_report_20260322_223919.docx	Code-generated summary artifact	Generated summary report preserved as emitted by the reporting code.
final_report_20260322_223919.docx	Code-generated final report artifact	Generated final report preserved as emitted by the reporting code.
detail.py	Raw-output generation script	Code path used to generate detail-level Word outputs.
planned_request_event_index_15552.csv	Denominator-only planned-event index	Machine-readable request positions and factor labels only; no observed outcomes are stored in this file.

## 4 Experimental Design, Materials and Methods

### 4.1 Factorial design construction

The benchmark design was constructed as the Cartesian product of three backend families, four runtime-packaging modes, two constraint settings, and two transport settings, yielding 48 deployment combinations. Each combination used the same 324 planned requests: 36 routed request instances were executed in each of three difficulty strata and three concurrency levels (36 × 3 × 3). The Simple stratum supplied clear route prototypes, Complex/Interrupt cases tested short history, interruption, and carry-over, and Edge Case prompts introduced lexical ambiguity, adversarial phrasing, or mixed-intent cues. Reusing the same pool across all combinations supports paired configuration comparison. Each row in data/design/full_factorial_design_48.csv has a stable combination_id so that the design, planned index, processed summaries, figure data, and manuscript tables can be joined without relying on row order.

### 4.2 Prompt pool, route ontology, and benchmark key

The route ontology was fixed before backend execution. The chat label covers conversational assistance and low-commitment interaction; task covers planning, scheduling, explicit work organization, or workflow execution; dev covers software development, implementation, and debugging; and doc covers document-grounded summarization, extraction, or transformation. State retention was scored only when an interruption or carry-over target existed; simple stateless cases were not included in the state-retention denominator.

Each benchmark prompt was created under one of the four route templates and carried a predefined expected route in a frozen benchmark key before any provider call. The same prompt pool and key were reused across all 48 combinations. Routing accuracy was computed by exact comparison between the emitted route and this predefined key, and the expected labels were not changed after model outputs were examined.

### 4.3 Original raw-data capture

The original generated-report package is F.zip. It contains code-generated Word/report outputs such as details_20260322_165132, detail_*.docx, summary_report_20260322_223919.docx, final_report_20260322_223919.docx, and detail.py. These objects are classified as original raw benchmark artifacts because they are the first persistent files emitted by the benchmark/reporting code before sanitization, aggregation, manuscript rewriting, or template conversion. They are not provider-native response payloads. The copies deposited in F.zip were archived as generated, without manual post-generation editing, and are assigned to data/raw/F.zip and data/raw/F_zip_original_raw_outputs/.

### 4.4 Raw-level planned request-event index

The file data/raw/planned_request_event_index_15552.csv is a design-only denominator index. Each row records raw_index_id, combination_id, backend, mode, constraint_setting, transport_setting, request_index_within_combination, prompt_pool_size, record_level, and sanitization_status. It does not contain expected or emitted route labels, provider responses, request-level correctness, latency, token counts, or failure classes. Its role is to verify the 15,552 planned request positions and connect them to the 48-row design; the observed aggregate metrics and Table 6 failure taxonomy were derived separately from the archived benchmark reports.

### 4.5 Runtime-package modes

The four mode labels encode package-level differences in serialization burden and the locus of final structure realization. MJ is the low-budget direct-JSON package, and SJ is the higher-budget direct-JSON package. MJS is a historical label associated with a streaming-compatible JSON package, but transport is modeled as an independent stream/non-stream factor overlaid on every named mode. MCLR requests a compact intermediate code and reconstructs the canonical JSON object with deterministic client logic. Mode and transport should therefore not be conflated.

### 4.6 Backend identities and execution snapshot

Table 8 records the exact backend strings and access modes recovered from the archived implementation. All three backends were accessed as hosted services; none was self-hosted. The generated report artifacts are timestamped 22 March 2026, and this date is reported as the benchmark snapshot date. No separate immutable provider-side snapshot identifier was retained in the archived materials.

**Table 8:** Backend model identifiers, hosted access modes, and archived benchmark snapshot.

Backend family	Exact model identifier and access mode	Archived snapshot note
OpenAI	gpt-4o; OpenAI hosted API (not self-hosted)	Report artifacts dated 22 March 2026; no additional immutable snapshot ID retained.
Gemini	gemini-3.1-flash-lite-preview; Google Gemini hosted API (not self-hosted)	Report artifacts dated 22 March 2026; no additional immutable snapshot ID retained.
Llama	llama-3.3-70b-versatile; Groq-compatible hosted API (not self-hosted)	Report artifacts dated 22 March 2026; no additional immutable snapshot ID retained.

The common harness used random seed 20260316, a 60-s request timeout, at most three retries, and a global limit of 10 requests per minute. Constrained calls used low-variance settings: temperature was 0.0 where supported and top_p was 0.1 where available. The Gemini limited profile used the provider's minimal-thinking setting; the Llama limited profile used temperature 0.0; and GPT-4o used the deterministic routing configuration retained in the harness. The archived reference implementation assigned nominal maximum output-token budgets of 100 for MJ, 1024 for SJ, 96 for MJS, and 30 for MCLR. These settings describe the archived package profiles and remain subject to provider-specific API support.

A complete dependency lockfile and exact historical SDK package versions were not retained in the released materials. The manuscript therefore reports the exact model strings, access modes, dated report snapshot, and recorded runtime controls without claiming a fully pinned provider environment.

### 4.7 Schema, validation, and processed metrics

The JSON schema in data/schema/request_level_event_schema.json documents the intended structure of a future fully sanitized request-level outcome record. It is not the schema of planned_request_event_index_15552.csv, whose released columns are limited to design and denominator fields. The schema follows JSON Schema Draft 2020-12 so that required outcome fields, enumerated factor labels, nullable observations, and numeric bounds can be checked with standard validators [11].

Metric generation used the same canonical scorer across all modes. For MJ, SJ, and MJS, format compliance required parseable JSON with the required fields; for MCLR, compliance was evaluated after deterministic local reconstruction into the same canonical object. Routing accuracy was exact agreement with the frozen route key. State retention was computed only for applicable interruption and carry-over cases. Latency used full-response time because downstream dispatch requires the complete control record, and token counts were summed within each processed cell. Failed requests were assigned one aggregate outcome class, producing the mutually exclusive counts reported in Table 6. These steps generated backend-mode means, effect tables, targeted contrasts, workflow-completion summaries, and the failure taxonomy.

### 4.8 Processed statistical-layer tables retained in Mendeley data

The processed statistical CSV files are retained under data/processed and mirrored under data/tables. They were derived from the archived benchmark reports rather than from the denominator-only planned index. The mirrored layout keeps the cell means, ANOVA effects, targeted contrasts, workflow-completion summaries, failure taxonomy, and manuscript-facing tables available through the Mendeley Data package without a separate data dependency.

### 4.9 Offline validation and integrity checks

The script code/reproduce_summary_tables.py is an offline validation entry point rather than a full re-execution of the hosted APIs. It checks that the design matrix has 48 rows, the planned-event index has 15,552 rows, the backend-mode summary has 12 cells, and the failure-taxonomy counts sum to the expected denominator. A recommended first-use sequence is to verify metadata/checksums_sha256.csv, inspect the design matrix, inspect the planned index and metric dictionary, run the validation script, and then open the source CSV associated with the required table or figure. Re-running the original backend calls requires separate provider credentials and current API access and may not reproduce the dated provider behavior.

Table 9 links the manuscript tables and figures to repository source files and identifies where the execution settings and revised workflow artwork are documented.

**Table 9:** Navigation index for manuscript tables, figures, execution settings, and supporting resources.

Manuscript object or setting	Repository file or article location	Use or verification role
Fig. 1: repository components	metadata/data_provenance_raw_vs_processed.md; Figure_1_Repository_Components.png (article artwork)	Shows the component relationships and the separation of planned positions from observed summaries. Revised artwork is supplied with this article.
Figure 2: backend-by-mode comparison	docs/figure_data/result_backend_mode_metrics_source.csv; docs/figures/result_backend_mode_metrics.png	Provides the 12-cell source data and the deposited result figure.
Table 1: package inventory	metadata/file_inventory.csv; data/tables/table_1_file_inventory_main.csv	Maps deposited objects to their roles and release paths.
Table 2: benchmark dimensions	data/design/full_factorial_design_48.csv; data/tables/table_2_benchmark_dimensions.csv	Verifies factor levels, 48 combinations, and the 15,552-position denominator.
Table 3: runtime packages	data/design/runtime_mode_definitions.csv; data/tables/table_3_runtime_mode_dictionary.csv	Defines MJ, SJ, MJS, and MCLR independently of the stream/non-stream transport factor.
Table 4: metric definitions	metadata/metric_dictionary.csv; data/tables/table_4_main_processed_metrics.csv	Defines the six released aggregate metrics and their interpretation.
Table 5: backend-by-mode cells	data/processed/backend_mode_cell_means.csv	Supplies all 12 backend-by-mode summary rows and descriptive half-width fields.
Table 6: failure taxonomy	data/processed/failure_taxonomy_overall.csv	Supplies the mutually exclusive aggregate outcome counts over the shared benchmark denominator.
Table 7: provenance boundary	metadata/data_provenance_raw_vs_processed.md; data/raw/README_raw_data.md	Explains generated report artifacts, the denominator-only planned index, sanitization, and non-released provider payloads.
Table 8 and key execution settings	Table 8 and adjacent methods text; docs/data_acquisition_protocol.md (general acquisition workflow)	Locates the reported model strings, access modes, snapshot date, and runtime controls. The protocol supplies general workflow context.
Offline validation and integrity	code/reproduce_summary_tables.py; metadata/checksums_sha256.csv	Checks design size, denominator, summary-cell count, failure totals, and file integrity without rerunning hosted APIs.
Reuse licences	LICENSE.txt	States CC BY 4.0 for data and metadata and MIT for reference code.

Fig. 1 is a schematic redrawn for this article; its artwork is supplied with the revised manuscript. Table 8 and the adjacent methods text report the execution settings, while the repository protocol describes the general acquisition workflow. These editorial revisions do not replace files in the cited Version 1 deposit.

### 4.10 Data sanitization

The package includes metadata/sanitization_notes.md and data/raw/README_raw_data.md. The public version retains the factorial design, denominator information, aggregate outcomes, figures, metadata, reusable schema documentation, and validation resources, but it does not redistribute API credentials, provider account metadata, non-public endpoint identifiers, private runtime secrets, private prompt content, or provider-native raw responses.

## Limitations

• The dataset is a dated hosted-API snapshot. The exact model strings and the 22 March 2026 report-artifact date are provided, but no immutable provider-side snapshot identifiers or complete dependency lockfile were retained. Provider behavior, APIs, model releases, pricing, and structured-output support may therefore differ in later runs.
• Version 1 remains the fixed historical benchmark snapshot dated 22 March 2026. Any future updates for backend or API compatibility will be issued as a new Mendeley Data version, with a changelog documenting the affected model/API identifiers, access dates, runtime settings, and modified scripts. The original Version 1 files and reported results will not be overwritten. Updates are conditional on a new release; continuous support for evolving hosted APIs and numerical equivalence across versions are not guaranteed.
• planned_request_event_index_15552.csv is a denominator-only design object rather than a request-level observed-outcome table. Provider-native payloads and account-level runtime metadata are not redistributed, so request-level reanalysis is bounded by the archived generated reports and the released aggregate tables.
• The public package supports inspection, offline validation, denominator verification, figure/table source tracing, and secondary aggregate analysis. Re-running the original backend calls requires separate credentials and current provider access and may not reproduce the historical provider snapshot.
• MCLR is a bundled package-level intervention combining compact emission, a low output budget, and deterministic local reconstruction. The Llama/MCLR result identifies a clear incompatibility regime, but the released benchmark does not isolate the marginal cause of each micro-design choice.
• The archived .docx files are first persistent author-generated report artifacts rather than provider-native response logs. Their reuse should respect this provenance boundary.

## Ethics Statement

The dataset contains programmatically generated benchmark events, raw code-generated software-benchmark reports, processed benchmark metadata, and scripts. It does not contain human-participant data, animal data, clinical data, personally identifiable information, identifiable private user records, animal experiments, or data collected from social media platforms. The authors have read and followed the ethical requirements for publication in Data in Brief. No ethics approval was required for the creation of these software-benchmark data objects.

## Credit Author Statement

**Hanlin Zhou:** Conceptualization, Methodology, Software, Data curation, Formal analysis, Validation, Visualization, Writing - original draft. **HuahYong Chan:** Conceptualization, Methodology, Supervision, Validation, Writing - review and editing.
